# Correlation of *MDR1* gene polymorphisms with anesthetic effect of sevoflurane–remifentanil following pediatric tonsillectomy

**DOI:** 10.1097/MD.0000000000007002

**Published:** 2017-06-16

**Authors:** Nian-Jun Shi, Wei-Xia Zhang, Ning Zhang, Li-Na Zhong, Ling-Ping Wang

**Affiliations:** Department of Anesthesiology, Linyi People's Hospital, Linyi, People's Republic of China.

**Keywords:** anesthetic effect, children, gene polymorphism, multidrug resistance gene 1, remifentanil, sevoflurane, tonsillectomy

## Abstract

**Background:**

The motive of this study was to investigate the collaboration between *MDR1* gene polymorphisms and anesthetic effects following pediatric tonsillectomy.

**Methods:**

All together 178 children undergoing tonsillectomy with preoperative sevoflurane–remifentanil anesthesia were selected. In order to determine *MDR1* gene polymorphisms of 3435C > T, 1236C > T, and 2677G > T/A, polymerase chain reaction–restriction fragment length polymorphism was used. Mean arterial pressure (MAP), diastolic blood pressure (DBP), systolic blood pressure (SBP), and heart rate (HR) at *T*_0_ (5 mins after the repose), *T*_1_ (0 min after tracheal intubation), *T*_2_ (5 mins after the tracheal intubation), *T*_3_ (0 min after the tonsillectomy), T4 (0 min after removal of the mouth-gag) and *T*_5_ (5 min after the extubation) were observed. The visual analog scale (VAS), the face, legs, activity, cry, and consolability (FLACC) pain assessment, and Ramsay sedation score were recorded after the patients gained consciousness. The adverse reactions were also observed.

**Results:**

As compared to the CT + TT genotype of MDR1 1236C > T, the time of induction, respiration recovery, eye-opening, and extubation of children with the CC genotype was found to be shorter (all *P* <.05); the MAP, SBP, DBP, and HR were significantly reduced at *T*_5_ in children that possessed the CC genotype (all *P* <.05), the VAS at postoperative 1, 2, 4, and 8 hours and Ramsay sedation score were decreased, while the FLACC score increased (all *P* <.05). It was found that the adverse reaction rate was lower in children bearing the CC genotype (*P* <.05).

**Conclusion:**

It could be concluded that anesthetic effect in patients with the MDR1 1236C > T CC genotype was found to be superior to those carrying the CT + TT genotype.

## 1 Introduction

The process of tonsillectomy is known to be one of the most common and severely painful surgeries that children undergo.^[1]^ The surgical procedure of tonsillectomy induces a number of postoperative morbidities, that includes nausea, vomiting, serious pain, and poor oral intake, as well as an emergence agitation, which may postpone the discharge and also undermine the patient's ability to do normal activities.^[2]^ Recently, along with the advancement in anesthetic and surgical techniques, tonsillectomy has become safer than old times.^[3]^ The 2 main drugs among many that are widely used for anesthetic purpose are known to be sevoflurane and remifentanil.^[4,5]^

Sevoflurane is one of the volatile anesthetics, which has low blood gas partition coefficient, aromatic odor, rapid onset, and low pungency into the airway.^[6]^ Because of this property, it is most widely applied during surgical operations and cesarean deliveries to induce and maintain pediatric anesthesia. However, emergence agitation induced by sevoflurane in children is the main drawback of this anesthetic with a reported incidence of over 80%.^[7]^ Remifentanil is a frequently used opioid that can decrease the sevoflurane requirement dose-dependency in children.^[8]^ The effect of remifentanil can be weakened quite fast and predictably after the cessation with no delay in restoration from the anesthesia.^[9]^ Nevertheless, it also has some drawbacks such as postoperative nausea and vomiting, muscle rigidity, and dose-dependent reductions in the heart rate (HR) as well as systolic blood pressure, and it also elevates postoperative analgesic requirement because of the acute opioid tolerance.^[10]^

MDR1, a known adenosine triphosphate-binding cassette from the subfamily B member 1 (ABCB1) gene, encodes a 170-kDa transmembrane protein, P-glycoprotein (P-gp). P-gp is an efflux pump for variety of lipophilic compounds.^[11,12]^ MDR1 is expressed in diverse tissues such as gastrointestinal and nasal respiratory mucosa, liver, kidneys, placenta, as well as the adrenal cortex.^[13]^ Till date many single-nucleotide polymorphisms (SNPs) have been identified in the exons of MDR1, among which, 1236C > T, 2677G > T/A, and 3435C > T in MDR1 are the main variants which are investigated repeatedly in different countries and races worldwide under multiple disease conditions.^[14–16]^ MDR1 is known to be an important transporter which constrains the accumulation of many drugs, such as chemotherapeutic and antiepileptic drugs.^[14]^

Evidence suggests that gene polymorphism may play an important role in drug anesthetic effect.^[17,18]^ Hence, our study aims to investigate the role of *MDR1* gene polymorphism of 1236C > T, 2677G > T/A, and 3435C > T in anesthetic effects of sevoflurane combined with remifentanil, so as to provide knowledge for better anesthetic effects on the pediatric tonsillectomy.

## 2 Material and methods

### 2.1 Subject and grouping

For the purpose of this study, 178 children out of which male and female were 100 and 78 respectively, subjected to receive tonsillectomy, based on general sevoflurane–remifentanil anesthesia, at Linyi People's Hospital from October 2013 to July 2016. All the children recruited for this study were aged between 3 and12 years with a mean age of 7.0 ± 2.4 years, and weighed around 14 to 44 kg with a mean weight of 29.7 ± 4.7 kg. The mean time taken by the operation performed was 44.9 ± 8.2 minutes, during which blood samples of all the children were successfully obtained in order to extract DNA. Children selected for this study were classified based on the American Society of Anesthesiologists physical status I or II.^[19]^

The involvement criteria for eligible patients were as follow:

1. People who underwent postoperative analgesia voluntarily.
2. People who had no allergy history to any kind of anesthetic drugs or no operation contraindication.

The noninvolvement criteria for noneligible patients were as follow:

1. Heart block or cardiac abnormalities.
2. Liver or renal dysfunction.
3. Drug abuse history
4. Allergy to any kind of drug used in this study.
5. Failure to intravenous injection.
6. Inability to cooperate.

This experiment was approved by the Ethics Committee of Linyi People's Hospital. Anesthesia informed consent was obtained from the parents or legal guardians.

### 2.2 Anesthesia

There was no administered during this experiment. After preoperative fasting of about 6 to 8 hours and without drug treatment before operation, all the subjects were examined for the vital signs that included electrocardiogram, HR, respiratory rate, and blood pressure. The effect of anesthesia began when all conditions were stable. Before the induction of anesthesia, the subjects were then intravenously injected with atropine of 0.1 mg/kg (Changle Pharmaceutical Co. Ltd, Xinxiel Co., Ltd, Xuzhou, Jiangsu, China), fentanyl of 2 μg/kg (Yichang Humanwell Pharmaceutical Co., Ltd, Yichang, Hubei, China), and midazolam of 0.1 mg/kg (Chemical Abstracts Service No., 96946-42-8; Yongnuo Pharmaceutical Co. Ltd, Yichang, Hubei, China). Then the anesthesia was induced with ventilation of 5% sevoflurane (Shanghai Hengrui Pharmaceutical Co. Ltd, Shanghai, China) in oxygen at 4 L/min. After all the patients had completely relaxed their muscles, tracheal intubation was performed. During the anesthesia process, all the subjects were given ventilation, mechanically to ensure the stable vital signs. Then 0.2 to 0.4 μg/kg/min of remifentanil (Yichang Humanwell Pharmaceutical Co., Ltd, Yichang, Hubei, China) was injected and sevoflurane was adjusted to 3% concentration to sustain anesthesia. Sevoflurane was discontinued after the operation. When the normal breathing, coughing, and swallowing function restored, the ventilator was removed. About 5 minutes after the oxygen supply was ceased, the percutaneous oxygen saturation (SPO_2_) was stabilized at about 95%. Patients were then shifted to the observation room.

### 2.3 Hemodynamics after anesthesia

The times taken for the following activities were recorded: time of anesthesia induction, eye-opening, respiration recovery, and extubation. Mean arterial pressure (MAP), diastolic blood pressure (DBP), systolic blood pressure (SBP), and HR of different genotypes were recorded and compared at 5 minutes after repose (*T*_0_), 0 minute after tracheal intubation (*T*_1_), 5 minutes after tracheal intubation (*T*_2_), 0 minute after tonsillectomy (*T*_3_), 0 minute after removal of mouth-gag (*T*_4_) and 5 minutes after extubation (*T*_5_).

### 2.4 Analgesia and adverse reaction observation

At postoperational 1, 2, 4, and 8 hours the visual analog scale (VAS) was recorded. The Ramsay sedation score 5, 15, and 30 minutes after extubation was assessed: 1 point for anxiousness or restlessness; 2 point for cooperativeness and tranquility; 3 point for responding to commands with unclear murmurs; 4 point for drowsiness and brisky response to calling; 5 point for drowsiness and sluggish response to calling; and 6 point for deep sleep or anesthesia.

A score of around 2 to 4 indicated an effective sedation while a score of around 5 to 6 indicated oversedation. The Face, Legs, Activity, Cry, and Consolability (FLACC) pain assessment 5, 15, and 30 minutes after extubation were as follows: 0 point for content and relaxed; 1 to 3 for slight pain; 4 to 6 for medium pain; and 7 to 10 for severe pain and uncomfortable. The postoperational adverse reaction rate followed by consciousness was also observed.

### 2.5 Peripheral blood sampling and DNA extraction

Fasting venous blood (3 mL) was sampled and placed into the sodium citrate anticoagulation tube, the tube walls of which were labeled with subject number and name. Using a DNA extraction kit (Article No., 52304; Qiagen Company, Hilden, Germany), the blood was centrifuged at 3000 rpm/min for 10 minutes to extract whole blood DNA from the peripheral blood, and DNA concentration detector (Model No., NANODROP2000; Thermo Fisher Scientific Inc., Waltham, MA) was applied in order to determine the DNA purity as well as DNA concentration at optical density with a wavelength of 260 nm. The concentration was set to 100 ± 20 ng/L and A260 nm/A280 nm ranged from1.6 to 1.8. The extracted DNA was then stored at a temperature of –40°C.

### 2.6 Detection of gene polymorphism

**Figure 1:**
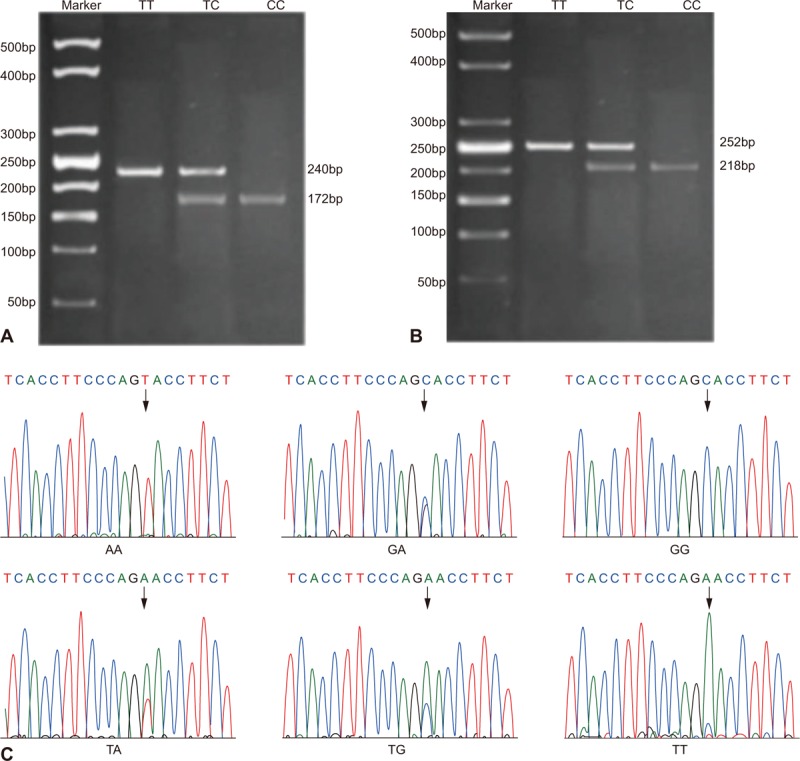
Electrophoretogram of *MDR1* gene enzyme digestion products. (A) MDR1 3435C > T products after enzyme digestion; lane 1, mutational homozygote TT; lane 2, mutational heterozygote TC; lane 3, wild homozygote CC. (B) MDR1 1236C > T products after enzyme digestion; lane 1, mutational homozygote TT; lane 2, mutational heterozygote TC; lane 3, wild homozygote CC. (C) Different genotypes of MDR12677 G > T/A after enzyme digestion.

The primer used in this experiment was synthesized by Nanjing Lejin Biotechnology Co. Ltd (Nanjing, China) and its sequence is listed in Table 1. The whole reaction system was 20 μL with extracted DNA of 1 μL, forward and reverse primer (10 pmol/mL) of 1 μL for each polymerase chain reaction (PCR) mixture of 10 μL, and deionized water of 7 μL (First Strand cDNA Synthesis Kit No., KP202-03; Tiangen Biotech Co. Ltd, Beijing, China). The reaction condition was predenaturation at a temperature of 95°C for 5 minutes and then the cycles began with denaturation at a temperature of 95°C for 30 seconds that annealed at 65°C for 40 seconds and elongated at 72°C for 7 minutes. Total of 40 cycles was performed. Eventually, elongation was performed at a temperature of 72°C for 10 minutes. After that, 3.5% agarose gel containing ethidium bromide of 0.5 mg/mL was mixed with amplification products of 4 μL and bromophenol blue sampling buffer solution of 1 μL. The mixed solution was then electrophoresed in 0.5 × Tris-Borate-EDTA buffer at 70 V for 15 minutes. In order to determine the success of the PCR, the amplification band was then observed under ultraviolet light and was also photographed. PCR products MDR11236 C > T and MDR13435 C > T were digested using restriction enzyme BsuR I (37°C) and Mbol (37°C), respectively. The restriction enzyme used for this purpose was purchased from New England Biolabs (Beijing) Ltd (Beijing, China). The procedure was strictly in allegiance with the given instructions. As G on MDR12677 G > T/A might mutate into A, gene genotyping was performed based on DNA sequencing using ABI-PRISM sequencer (Model No., 3730; Thermo Fisher Scientific Inc., Waltham, MA). The products of enzyme digestion were electrophoresed in 3.5% agarose gel at 120 V for 40 minutes and then observed under ultraviolet condition for the band (Fig. 1A and B ). The sequencing of MDR12677 G > T/A is noted down in Fig. 1C. In order to confirm the PCR results, 10% samples were randomly selected to perform bidirectional sequencing at Sangon Biotech Co. Ltd (Shanghai, China).

**Table 1:**
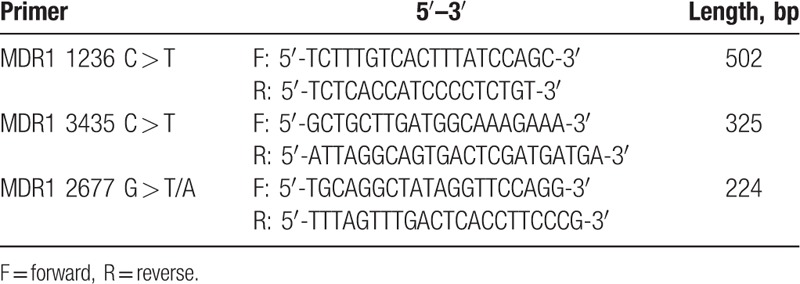
Prime sequences of MDR1 1236 C > T, 3435 C > T, and 2677 G > T/A.

### 2.7 Statistical analysis

Statistical analysis was performed using SPSS 19.0 (SPSS Inc., Chicago, IL). Continuous data consistent with normal distribution were demonstrated by mean ± standard deviation (SD). Homogeneity of variance was used to compare differences among multiple groups and the Student–Newman–Keuls *Q*-test was used to compare differences between 2 groups. Continuous data inconsistent with normal distribution was demonstrated by median. The Kruskal–Wallis *H*-test was held to have an inspection on comparisons between groups and the differences between 2 groups were then compared by the Nemenyi test. MDR1 haplotype was analyzed by Haplo.Stats1.4.3 based on the R software. Enumeration data was analyzed by the χ^2^ test with an inspection level at 0.05. All *P* values indicated 2-tailed probability. And *P* <.05 was considered to be statistically significant.

## 1 Results

### 1.1 Allele frequencies of MDR1 3435C > T, MDR1 1236C > T, and MDR1 2677G > T/A

A total of 178 subjects were recruited for the purpose of this study. According to the enzyme digestion products, the number of patients bearing MDR1 3435C > TMDR1 3435C > T wild homozygote CC, heterozygote CT, and mutational homozygote TT were 68, 80, and 30, respectively, and the mutation frequency of T allele was about 39%. The number of patients bearing MDR1 1236C > T wild homozygote CC, heterozygote CT, and the mutational homozygote TT were 19, 87, and 72, respectively, with the mutation frequency of T allele being at about 65%. The number of MDR1 2677G > T/A mutational heterozygote TG, GA, and TA were 75, 23, and 22, respectively. The number of mutational homozygote TT, GG, and AA were 23, 31, and 4, respectively. The mutation frequency of T allele was at a percentage of 40. The MDR1 gene distribution of 3435C > T, 1236C > T, and 2677G > T/A was consistent with Hardy–Weinberg equilibrium (all *P* >.05), which indicated that the gene distribution of MDR1 3435C > T, 1236C > T, and 2677G > T/A achieved genetic equilibrium, and it was then recorded and represented (Table 2).

**Table 2:**
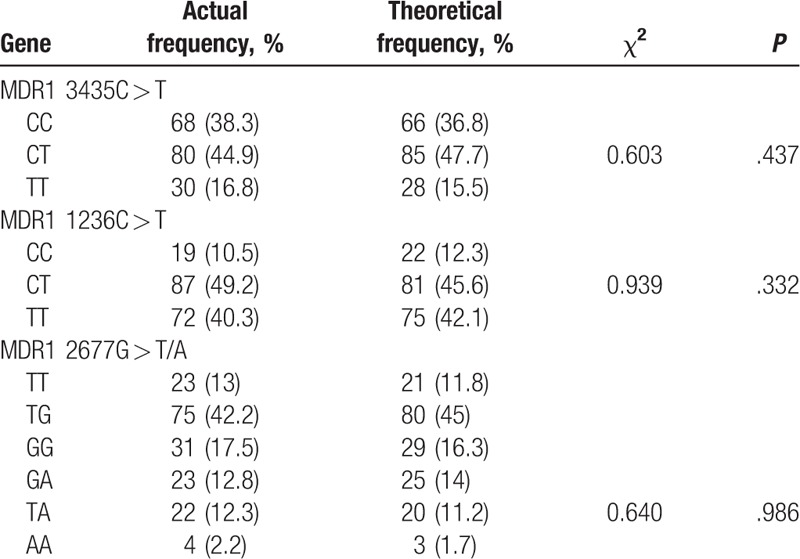
Hardy–Weinberg equilibrium of MDR1 1236 C > T, 3435 C > T, and 2677 G > T/A.

### 1.2 Effect of *MDR1* gene polymorphisms on anesthesia induction and restoration

As demonstrated in Table 3, there was no significant difference revealed between MDR1 3435C > T CC and CT + TT genotypes that concerned the time of induction, respiration recovery, eye-opening, and extubation (all *P* >.05). Compared to the MDR1 1236C > T CT + TT genotype, children bearing the CC genotype exhibited shorter times of induction, respiration recovery, eye-opening, and extubation (all *P* <.05). No significant differences were found between different genotypes on MDR1 2677G > T/A concerning the time of induction, respiration recovery, eye-opening, and the extubation (all *P* >.05).

**Table 3:**
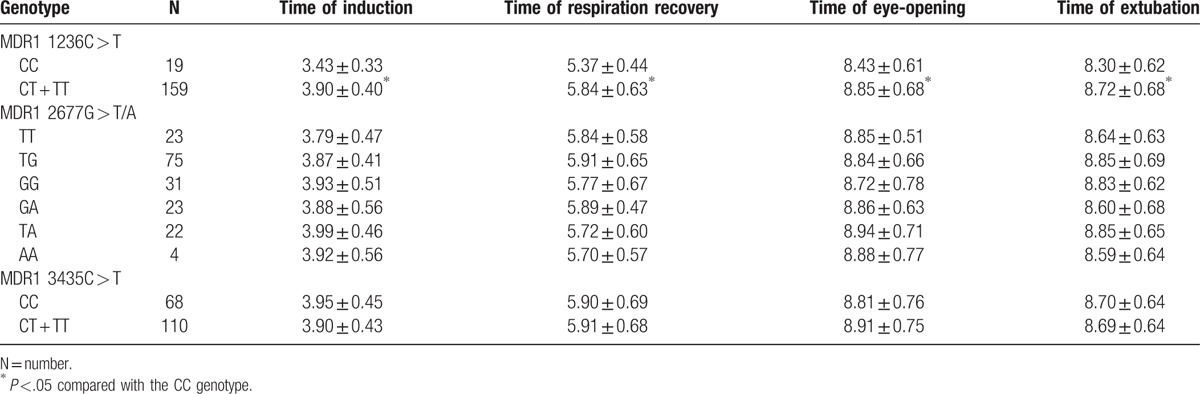
Effect of MDR1 gene polymorphisms of 1236 C > T, 3435 C > T, and 2677 G > T/A on anesthesia induction and restoration.

### 1.3 Effect of *MDR1* gene polymorphisms on hemodynamics after extubation

The MAP, SBP, DBP, and HR of patients with different genotypes at T1, T2, T3, T4, and T5 were found to be lower than those at *T*_0_ (all *P* <.05). There was no significant difference revealed between MDR1 3435C > T CC and CT + TT genotypes which concerned the MAP, SBP, DBP, and HR at *T*_0_, *T*_1_, *T*_2_, *T*_3_, *T*_4_, and *T*_5_ (all *P* >.05), which was also applicable for the different genotypes on MDR1 2677G > T/A (all *P* >.05). Compared to the MDR1 1236C > T CT + TT genotype, the children bearing the CC genotype exhibited significantly reduced MAP, SBP, DBP, and HR at T5 (all *P* <.05), and insignificant difference during other time points (all *P* >.05, Table 4).

**Table 4:**
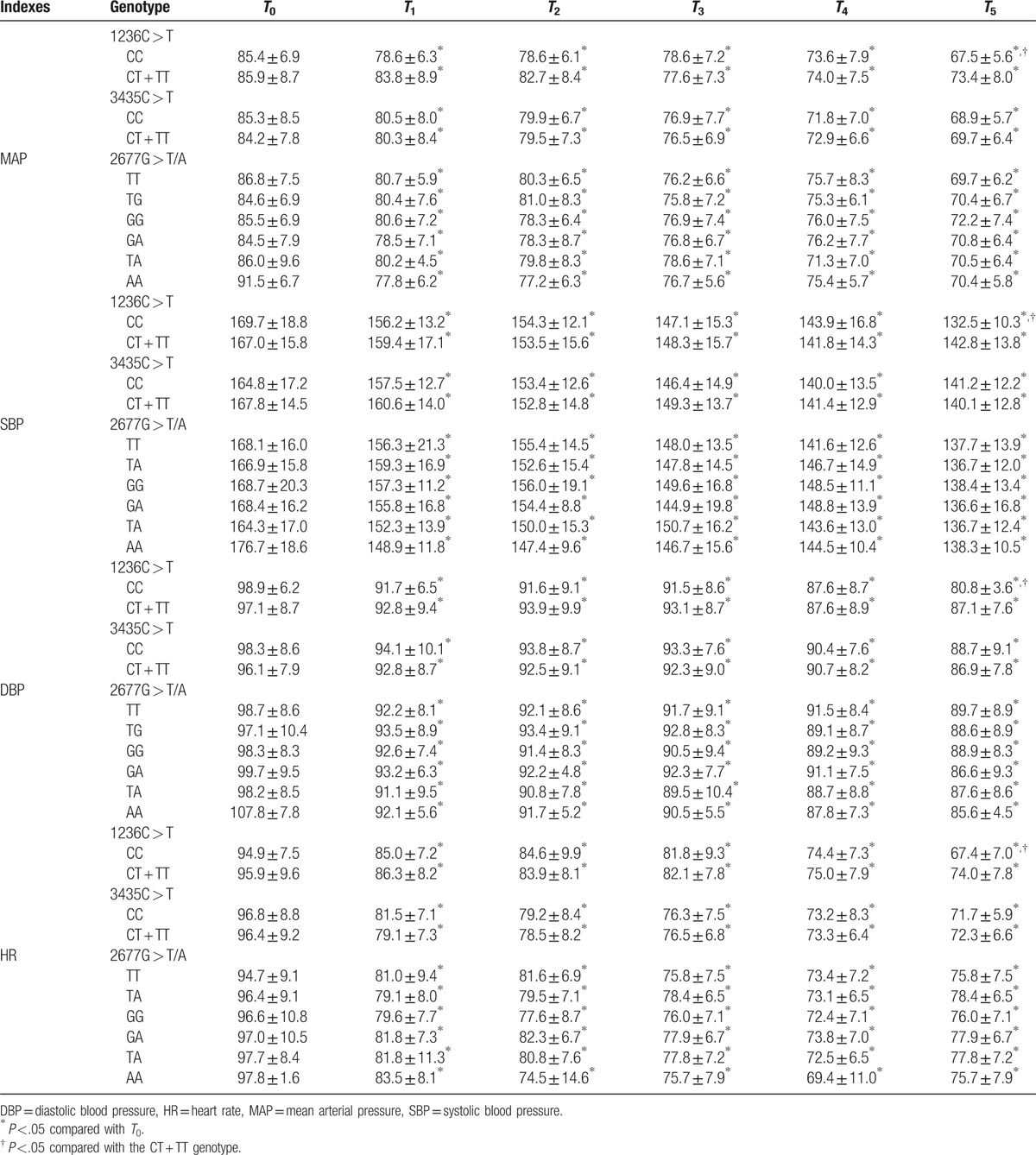
Effect of MDR1 gene polymorphisms of 1236 C > T, 3435 C > T, and 2677 G > T/A on MAP (mmHg), SBP (mmHg), DBP (mmHg), and HR (beat/min).

### 1.4 Effect of *MDR1* gene polymorphisms on VAS assessment

As compared to VAS at postoperative 1 hour, reduced VAS at postoperative 8 hours was found in all the genotypes. There was no significant difference revealed between MDR1 3435C > T CC and CT + TT genotypes in terms of VAS at each time point (all *P* >.05). Also, there was no significant difference observed among the different genotypes on MDR1 2677G > T/A (all *P* >.05). And compared with the MDR1 1236C > T CT + TT genotype, decreased VAS was indicated in children who suffered the CC genotype at postoperative 1, 2, 4, and 8 hours (all *P* <.05, Table 5).

**Table 5:**
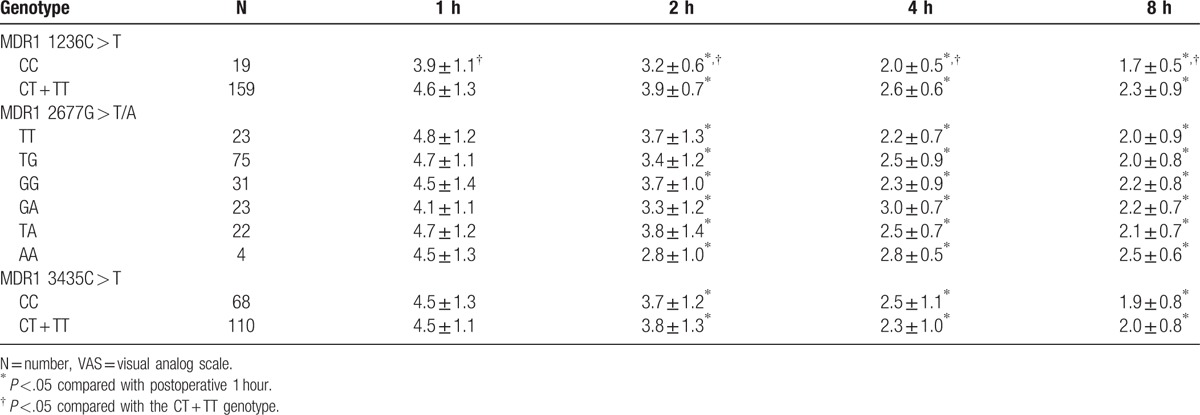
Effect of MDR1 gene polymorphisms of 1236 C > T, 3435 C > T, and 2677 G > T/A on VAS assessment.

### 1.5 Effect of *MDR1* gene polymorphisms on Ramsay sedation score and FLACC assessment

As shown in Table 6, there was no significant difference revealed between MDR1 3435C > T CC and CT + TT genotypes regarding the Ramsay sedation scores and FLACC scores after extubation for 5, 10, and 30 minutes (all *P* >.05). No significant differences were observed among different genotypes on MDR1 2677G > T/A (all *P* >.05). In comparison with the MDR1 1236C > T CT + TT genotype, Ramsay scores at the 3 time points were reduced in children with the CC genotype, while FLACC scores were found to be higher (all *P* <.05).

**Table 6:**
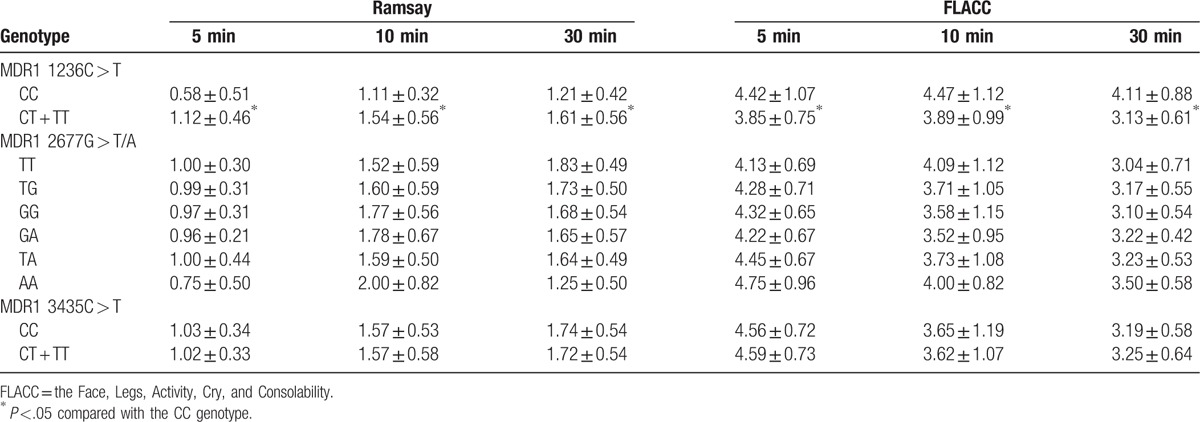
Effect of MDR1 gene polymorphisms of 1236 C > T, 3435 C > T ,and 2677 G > T/A on Ramsay sedation score and FLACC assessment.

### 1.6 Effect of *MDR1* gene polymorphisms on adverse reaction rate following consciousness

After tonsillectomy, there will be common adverse reactions experienced by patients that include nausea, vomiting, agitation, breathing difficulty, severe cough, and laryngospasm. As shown in Table 7, there was no significant difference revealed between MDR1 3435C > T CC and CT + TT genotypes regarding the adverse reaction rate (all *P* >.05). The comparisons of adverse reaction rate among different genotypes on MDR1 2677G > T/A were also found to be insignificant (all *P* >.05). In comparison with the MDR1 1236C > T CT + TT genotype, the patients bearing the CC genotype had lower adverse reaction rate (all *P *<.05).

**Table 7:**
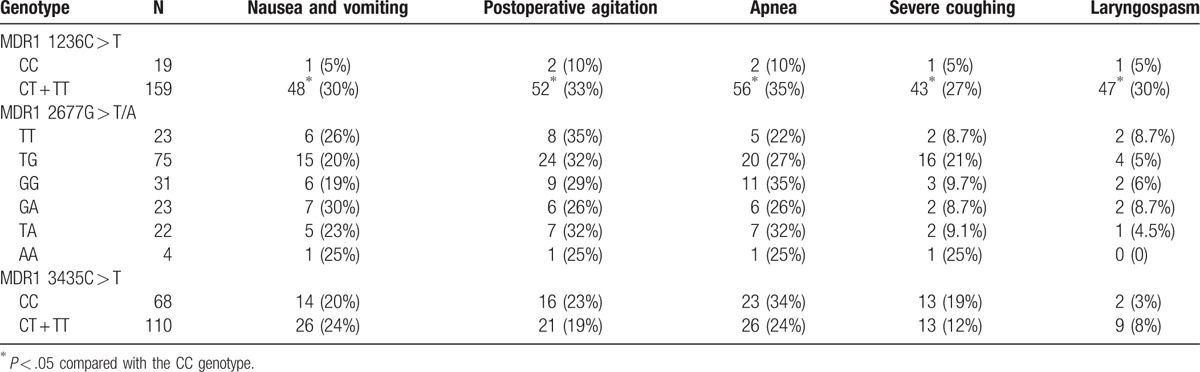
Effect of MDR1 gene polymorphisms of 1236 C > T, 3435 C > T and 2677 G > T/A on adverse reaction rate following consciousness (%).

## 2 Discussion

Lately, it is indicating that gene polymorphism could play an important role in drug anesthetic or analgesic effect.^[20,21]^ As known, MDR1 is an important transporter which can constrain the accumulation of chemotherapeutic drugs.^[14]^ Consequently, the role of *MDR1* gene polymorphism of 1236C > T, 2677G > T/A, and 3435C > T in the anesthetic effects of the drug sevoflurane combined with remifentanil was investigated through this study. According to the results obtained, it was revealed that SNPs of 1236C > T contributed to an individual variation in anesthetic effects that follow the pediatric tonsillectomy, and also it was figured out that the patients with the MDR1 1236C > T CC genotype displayed superior anesthetic effects compared to those patients who possessed the CT + TT genotype.

Firstly, the study data assured that in comparison to the MDR1 1236C > T CT + TT genotype, the children bearing the CC genotype exhibited shorter times of induction, respiration recovery, eye-opening, and extubation. MDR1 is something that can be found in variety of tissues including gastrointestinal and nasal respiratory mucosa, liver, kidneys, placenta, as well as in the adrenal cortex.^[13]^ According to many studies, it is known that the sevoflurane–remifentanil drug agents have been extensively applied for anesthesia, analgesia, and also procedural sedation.^[22,23]^ Recovery time of self-respiration, eye-opening time on verbal command, and extubation time were taken into consideration and compared for anesthetic effects, following the cessation of anesthetic agents.^[24]^

The study has also revealed that in comparison to the MDR1 1236C > T CT + TT genotype, children bearing CC genotype displayed significantly reduced MAP, SBP, DBP, and HR at *T*_5_, decreased VAS and Ramsay sedation scores, as well as increased FLACC score. Additionally, the lower adverse reaction rate following tonsillectomy was also demonstrated in the MDR1 1236C > T CC genotype compared with the CT + TT genotype. Using MAP, SBP, DBP, and HR, often the hemodynamic stability during anesthesia is measured.^[25]^ VAS, Ramsay, and FLACC scores are used to assess the pain scales after the emergence of an anesthesia.^[26,27]^ Many kinds of known adverse effects, such as vomiting, agitation, laryngospasm, severe coughing, and so on might be caused by the drugs sevoflurane–remifentanil anesthesia and tracheal extubation.^[2,28]^

A critical impact on the therapeutic efficacy and the pharmacokinetics of various drugs are displayed by alterations in the expressions and the activities of MDR1-encoded P-gp.^[14]^ Because of this reason, the P-gp levels can influence the entrance of drugs into the cells.^[13]^ Evidence suggests that elevated levels and enhanced activity of P-gp are conferred more by the CC genotype, whereas individuals who bear the TT genotype seem to have decreased P-gp activity.^[11]^ Genetic causes of MDR1 for the anesthetic effects are varied.^[11,29]^ According to the reports by Qi and his colleagues, MDR1 genetic variants rs12720464 and rs1055302 account for the individual variation of time course of action in patients undergoing general anesthesia with a single dose of rocuronium.^[30]^ Sadhasivam et al^[1]^ indicate that children who possess GG and GA genotypes of MDR1 rs9282564 present higher risks of opioid-related respiratory depression that leads to prolonged hospitalization and 1 copy of the minor allele (G) elevates the odds of prolonged stay owing to postoperative respiratory depression. The experiment held by Sia et al^[31]^ connects MDR1 1236C > T, 2677G > T/A, and 3435C > T to the chronic pain in women receiving spinal anesthesia with intrathecal morphine for elective caesarean section; and they reveal no statistical difference in the total consumption of morphine, side effects, and pain scores among different genotypes. Whereas women with 3435C > T allele demonstrated a higher risk of suffering with persistent postoperative pain. Farhat et al's ^[32]^ experiment indicated that management nausea and vomiting under intravenous administration of ondansetron are all related to the MDR1 2677G > T/A TT genotype. It is further proved that 1236C > T SNPs of MDR1 gene may be useful for predicting antiemetic response for ondansetron,^[29]^ which is quite similar to our findings. Despite the aforementioned studies, our study confirmed that there is no significant association between SNPs of 2677G > T/A and 3435C > T that concern anesthesia induction, emergence from anesthesia, hemodynamic changes, pain assessment, or adverse reactions.

In conclusion, it can be said that our study work gives evidence according to which SNPs of MDR1 1236C > T are partly the reasons for single differences in the anesthetic effects that follow pediatric tonsillectomy and that children who possess the MDR1 1236C > T CC genotype prove to have superior anesthetic effects as compared to those children who possess the CT + TT genotype in terms of anesthetic induction and restoration, hemodynamic changes, pain and sedation assessment, and also adverse reactions. Through this study, the possible underlying mechanism for the effects anesthetic by the drugs sevoflurane–remifentanil were explored. Nevertheless, the agenda of the study was limited due to the following reasons: first, because the complex that underlay the functions and interactions of gene polymorphisms restricted us from conducting experiments regarding other target genes. And the second reason is that our time, budget as well as research population were limited. Hence, further analysis of interactions between varieties of gene polymorphisms with larger populations was a novel target to be investigated and studied on.

## Acknowledgment

The authors want to show their appreciation to reviewers for their helpful comments.
